# Effect of Nd:YAG Laser with/without Graphite Coating on Bonding of Lithium Disilicate Glass-Ceramic to Human Dentin

**DOI:** 10.1155/2021/6677159

**Published:** 2021-03-15

**Authors:** Amjad Abu Hasna, Stephanie Semmelmann, Fernanda Alves Feitosa, Danilo De Souza Andrade, Franklin R Tay, Cesar Rogério Pucci

**Affiliations:** ^1^Department of Restorative Dentistry, Endodontics Division, Institute of Science and Technology, Sa˜o Paulo State University (UNESP), Sa˜o José dos Campos, SP, Brazil; ^2^Department of Restorative Dentistry, Institute of Science and Technology, Sa˜o Paulo State University (UNESP), Sa˜o José dos Campos, SP, Brazil; ^3^São Lucas University, Avenida Da Saudade 26 Caçapava, SP, Brazil; ^4^Department of Endodontics, The Dental College of Georgia, Augusta University, Augusta, GA, USA

## Abstract

This study evaluated the effect of different surface treatments on the tensile bond strength between lithium disilicate glass-ceramics, resin cement, and dentin. Fifty truncated cone-shape glass-ceramics were divided into five groups (*n* = 10): G1, control: 10% hydrofluoric acid (HF); G2, Nd:YAG laser + silane; G3, Sil + Nd:YAG laser; G4, graphite + Nd:YAG laser + Sil; and G5, graphite + Sil + Nd:YAG laser. Fifty human third-molars were cut to cylindrical shape and polished to standardize the bonding surfaces. The glass-ceramic specimens were bonded to dentin with a dual-cured resin cement and stored in distilled water for 24 h at 37ºC. Tensile testing was performed on a universal testing machine (10 Kgf load cell at 1 mm/min) until failure. The bond strength values (mean ± SD) in MPa were G1 (9.4 ± 2.3), G2 (9.7 ± 2.0), G3 (6.7 ± 1.9), G4 (4.6 ± 1.1), and G5 (1.2 ± 0.3). Nd:YAG laser and HF improve the bond strength between lithium disilicate glass-ceramics, resin cement, and dentin. The application of a graphite layer prior to Nd:YAG laser irradiation negatively affects this bonding and presented inferior results.

## 1. Introduction

Glass-ceramics were introduced into dentistry as early as 1885 [1] and have improved substantially since then. Despite their esthetic advantages, glass-ceramics are brittle and highly susceptible to fracture [2]. To date, glass-ceramics have improved significantly in their mechanical properties [3]. There is an increasing tendency to use lithium disilicate glass-ceramics in restorative dentistry because of its combined esthetic values, optimal mechanical properties, and excellent optical properties [4,5].

Different surface treatment techniques have been proposed for improving the bond strength between silicate-based glass-ceramics and resin cements. Hydrofluoric acid (HF) etching is the most commonly used for conditioning silicate-based glass-ceramic surfaces [6]. As well, neodymium-doped yttrium aluminum garnet (Nd:YAG) lasers are used to increase the roughness of glass-ceramic surfaces and improve their adhesion to resin cements [7,8]. This is achieved through the creation of microporosities, increase in surface energy, and improved wetting by the resin cement [8].

The wavelength of Nd:YAG laser is 1064 nm, which is in the invisible nonionizing infrared range. Emission in the pulsed mode is well absorbed by pigmented chromophores, present in soft tissues. Because absorption by hard dental tissues is very limited, clinical procedures involving the use of Nd:YAG lasers may be performed in the vicinity of enamel, dentin, and cementum without creating undue thermal damage [9].

The rationale of Nd:YAG laser irradiation of a glass-ceramic surface is to increase the irregularity of the glass-ceramic-cement interface to augment the surface energy and facilitate silane application for durable resin-glass-ceramic bonding [10]. Because the glass-ceramic substrate is water-free and its color is opaque white, the glass-ceramic surface may not absorb the emitted Nd:YAG laser energy sufficiently [11].

The application of a coating layer on the glass-ceramic surface has been proposed as a method to increase laser energy absorption. Graphite is a material that has high absorptivity and has been recommended as a coating material for increasing laser absorption [12]. However, sometimes it presents poor outcomes in bonding improvements [13].

The increasing demands of all-glass-ceramic restorations alert the relevance of testing of different surface treatments like Nd:YAG laser and graphite and their combined effect on the bond strength glass-ceramic materials and resin cements applied to human dentin and this was the objective of this study. The null hypothesis tested was that Nd:YAG laser irradiation and graphite coating of the glass-ceramic surface have no effect on improving the bond strength between resin cement and lithium disilicate glass-ceramic.

## 2. Material and Methods

### 2.1. Pressed Glass-Ceramic Specimen Preparation

Fifty lithium disilicate truncated cones (IPS e.max Press; Ivoclar-Vivadent, Schaan, Lichtenstein) were fabricated using a lost-wax technique. Low contraction wax (Renfert Geo; Renfert GmbH, Hilzingen, Germany) was poured into a 4 mm thick metal split-mold with a 2 mm diameter wide base and a 4 mm diameter wide top surface [14]. All the specimens were heat-pressed according to the manufacturer's instructions and then wet-polished with 600-grit silicon carbide paper in a polisher (DP-10; Panambra, São Paulo, SP, Brazil) utilizing running water to dissipate the heat generated during polishing. The polished specimens were immersed in an ultrasonic bath for 5 min to clean polishing remnants. They were randomly divided into five groups (*N* = 10):

I. Control, hydrofluoric acid: 10% HF (Dentsply DeTrey) was used to etch the specimens for 30 s. The same time was used to rinse the specimens with a water jet. A silane (Monobond Plus, Ivoclar-Vivadent, Schaan, Lichtenstein) was applied to the cleaned, etched surface for 30 s, after which the silane air-dried for 30 s.

II. Nd:YAG laser + Sil: Each specimen was irradiated with Nd:YAG laser (Pulse Master 600 IQ; American Dental Technologies Inc., Corpus Christi, TX, USA) with an energy output of 120 mJ. The pulse repetition rate was set at 15 pps and a 320 µm diameter laser optical fiber was placed at 12 mm away from the specimen surface for 1 min with water spray cooling (5 sec). The irradiated glass-ceramic surfaces were then etched with HF and the silane was applied following the same protocol used in the control group.

III. Sil + Nd:YAG laser: The specimens were etched with HF and the silane was applied as in the control prior to laser irradiation which was performed using the same parameters described for the Nd:YAG laser group.

IV. Graphite + Nd:YAG laser + Sil: Each specimen surface was coated with graphite prior to laser irradiation using the same parameters described for the Nd:YAG laser group. The same protocol was used to etch (HF) and prime (silane) over the surfaces treated by graphite and laser.

V. Graphite + Sil + Nd:YAG laser: The glass-ceramic surfaces were etched with HF for 30 s, rinsed with water spray for 30 s, coated with graphite, primed with silane, air-dried, and irradiated with Nd:YAG laser using the same parameters described for the Nd:YAG laser group.

### 2.2. Graphite Coating

The glass-ceramic surfaces were coated directly with fine grain (particle size: 5–25 *μ*m) graphite powder (Pressol, Nuremberg, Germany) without a previous manipulation forming a slightly thin layer (approximately 30 ± 5 *μ*m). As the applied area can be identified, care was taken not to apply two layers. This thickness was confirmed posteriorly with scanning electron microscopy. At the end of the irradiations, samples were carefully rinsed with distilled water in order to eliminate the residual graphite.

### 2.3. Dentin Specimen Preparation

Fifty human third molars were obtained from patients who were scheduled to have those teeth removed as part of their treatment plan. The protocol for the use of human teeth for benchtop research was approved by the Research Ethics Committee Involving Human Beings, Institute of Science and Technology, Sã˜o Paulo State University (UNESP) (No. 874675) with informed consent obtained from the donating subjects with respect to the use of human tissues. This work was performed in accordance with the Code of Ethics of the World Medical Association Declaration of Helsinki for experiments involving humans.

Decayed teeth or restored teeth were excluded from the study. All the molars had their occlusal surface abraded in the DP-10 polishing machine, using 400-grit silicon carbide paper under water cooling, until dentin was exposed. The teeth were embedded in chemically cured acrylic resin. A 2 mm diameter dentin bonding surface was created using a diamond-coated trephine drill (2 mm internal diameter) to match the diameter of the lithium disilicate truncated cone to be bonded. The dentin surface was polished with 600 grit silicon carbide paper to standardize the surfaces. Each tooth was indented under constant water cooling. The dentin surface had no pulp cavity and this was confirmed radiographically.

### 2.4. Bonding Procedures

The acrylic resin-embedded dentin specimens were conditioned and prepared for cementation following the manufacturer's recommendations. Each dentin surface was conditioned with 37% phosphoric acid for 15 s. This was followed by copious water-rinsing for 15 seconds. The surface was briefly blown-dry. ScotchBond Universal (3M ESPE) dentin adhesive was applied to the etched dentin for 20 s, dried for 10 s, and light-cured for 10 s using a light-emitting diode (LED) light-curing unit (Radii-Cal LED; SDI, Bayswater, Victoria, Australia) with an energy output of 800 mW/cm^2^.

Each glass-ceramic specimen was bonded to the adhesive-coated dentin using a dual-cured resin cement (Variolink II; Ivoclar-Vivadent, Schaan, Lichtenstein). The resin cement was light-cured for 40 s directly at the interface between the glass-ceramic and dentin specimens.

### 2.5. Tensile Bond Strength

**Figure 1:**
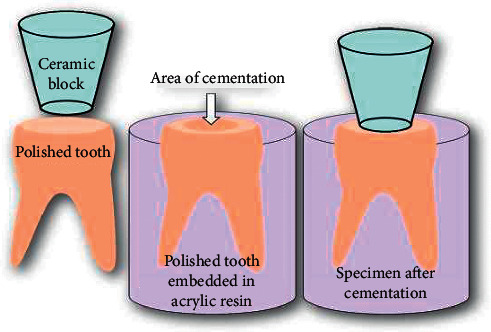
Schematic illustration of specimen preparation (dentin, resin cement, and glass-ceramic).

After cementation, the specimens as seen in Figure 1 were stored in distilled water at 37°C for 24 h until ensuring complete polymerization of the resin cement. Tensile testing was subsequently performed using a universal testing machine (EMIC-2000. EMIC, São José dos Pinhais, SP, Brazil) using a crosshead speed of 1.0 mm/min and with a 10 Kgf load cell. Loading was performed in tension until failure. The maximum force value was recorded for the calculation of tensile bond strength (in MPa).

### 2.6. Scanning Electron Microscopy

After bond testing, representative specimens derived from the separated glass-ceramic side of the assembly were dehydrated, sputter-coated with gold-palladium, and examined with a scanning electron microscope (SEM; Inspect S50, FEI Company, Hillsboro, OR, USA) operated at 15 kV.

### 2.7. Failure Mode Analysis

Qualitative analysis was performed with stereomicroscopy (Discovery V20, Germany) at 20× magnification for failure mode analysis of each specimen as the following:

1. Adhesive failure in dentin
2. Adhesive failure in resin cement
3. Adhesive failure in glass-ceramics
4. Adhesive failure in the glass-ceramic/resin cement interface
5. Cohesive failure in cement
6. Mixed failure

### 2.8. Statistical Analysis

Tensile testing data were analyzed for their normality (Shapiro–Wilk test) and homoscedasticity assumptions (modified Levene test). Because the data did not violate the assumptions for parametric statistical testing, they were analyzed using one-way analysis of variance. Post-hoc pairwise comparisons were conducted using the Tukey test. For all analyses, statistical significance was preset at *α* = 0.05. All statistical analyses were performed by GraphPad Prism 6 (La Jolla, CA, USA).

## 3. Results

There was no statistical difference between the control group and the Nd:YAG laser + Sil group (Table 1). These two groups had the highest bond strength compared with the other three groups and there was a significant difference when compared to the other tested groups.

Tensile bond strength values were in the order: control = Nd:YAG laser + Sil > Sil + Nd:YAG laser > graphite + Sil + Nd:YAG laser > graphite + Nd:YAG laser + Sil (*p*=0,001).

**Figure 2:**
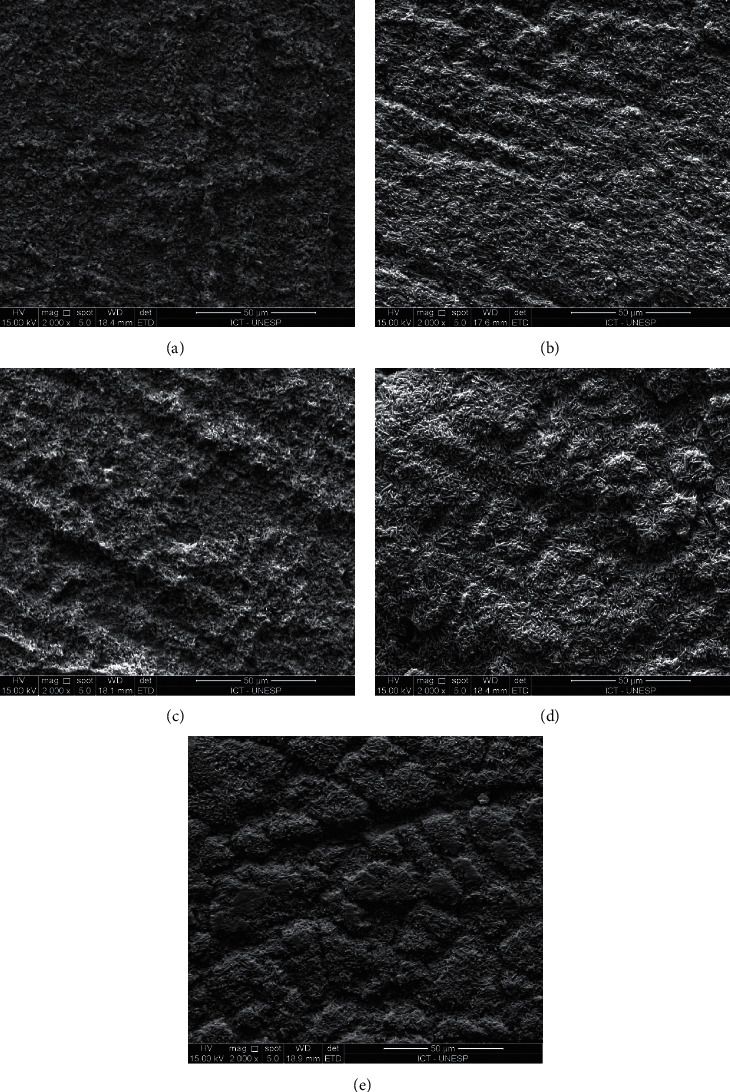
Representative scanning electron microscopy images of the fractured glass-ceramic side of specimens that have been stressed to failure under tension. At high magnification (2,000×) lithium disilicate crystallites created by hydrofluoric acid etching could be seen after the resin cement was dislodged from the bonded interface. (a) Control (2,000X). The lithium disilicate crystallites created by hydrofluoric acid etching. (b) Nd:YAG laser + Sil group: greater roughness can be seen compared to the control group, and it is related to the laser application. (c) Sil + Nd:YAG laser group: the lithium disilicates crystallites created by the laser application. (d) Graphite + Sil + Nd:YAG laser group: cluster-like lithium disilicates crystallites formed by the increased absorbance of the laser caused by the presence of the graphite. (e) Graphite + Nd:YAG laser + Sil group: demonstrating a great amount of deep scratches showing a great loss of the structure of the ceramic and thus did not improve the bond strength.

All failures occurred within the resin cement or along the glass-ceramic-resin cement interface in all groups. There was no evidence of mixed failures that involve either the dentin or the glass-ceramic surface (Figure 2). At high magnification, exposed lithium silicate crystallites could be seen along the exposed glass-ceramic surface. This increase in surface roughness is attributed to HF etching [14].

## 4. Discussion

In light of the increasing use of lithium disilicate glass-ceramic in restorative dentistry, many studies have attempted to improve bonding of the glass-ceramic to methacrylate resin-based luting cements by altering the glass-ceramic surface with a Nd:YAG or an Er:YAG laser [13,15]. Others have examined the use of a graphite surface coating to improve the absorption of laser energy [16]. Different sandblasting techniques as well as different types of silanes have also been used to increase retention and clean and prime the glass-ceramic surfaces [17–19]. The rationale of all these proposals is to create microretentions on the glass-ceramic surface that improves bond strength [20].

The use of HF etching for enhancing the bonding of lithium disilicate glass-ceramics to dentin cannot be overemphasized. Mallikarjuna et al. used 9.6% HF to etch the intaglio surface of the lithium disilicate glass-ceramic for 1 min [21]. In the present study, 10% HF was used for 30 s and reasonable results were obtained for the control group that was not significantly different from specimens that were treated with Nd:YAG laser and silane. Another study has reported that 10% HF improves the adhesion of lithium disilicate to BisGMA/TEGDMA resin cement-luted dentin [22]. Even more, 5% HF etching for 30 s improves zirconia-reinforced lithium silicate ceramics adhesive bond strength [23].

The fact that Nd:YAG laser treatment has no significant difference of HF acid makes the justification of such study useless; however, it should be noted that HF acid negatively affects the fatigue behavior of glass-ceramics [24] and this explains the necessity of a one-step ceramic primer [25] or another surface treatment like a laser to obtain improved bonding [13].

Viskic et al. evaluated the effect of Nd:YAG and Er:YAG lasers on the surface roughness of glazed-press lithium disilicate glass-ceramic discs using scanning electron microscopy [26]. Both lasers did not result in adequate surface modification for bonding of orthodontic brackets on glazed lithium disilicate glass-ceramics. However, the control group in that study that was treated with 9.5% HF improved bonding by creating a homogeneously rough pattern of exposed glass-ceramic crystals. The same results were obtained by Liu et al. who evaluated the shear bond strength of zirconia glass-ceramics after irradiation with three output powers (1, 2, or 3 W) and three irradiation times (30, 60, or 90 s) [27]. The authors concluded that irradiation of zirconia glass-ceramics by Nd:YAG laser does not improve its surface properties and does not improve bond strength. Conversely, Kasraei et al. reported that irradiation of glass-ceramic surface by Nd:YAG laser improves its bonding durability to resin cement [28]. These results somehow agree with the outcomes of the present study, in which the Nd:YAG laser + Sil group was effective as HF acid without a significant statistical difference between both groups.

In the present study, the energy intensity of the Nd:YAG laser was 120 mJ. The selection of this energy intensity was based on the study by Andrade et al. [15]. In that study, the authors compared the effect of Nd:YAG laser irradiation using 80, 100, 120, and 140 mJ on bond strength of glass-ceramics and reported the best results using 120 mJ.

The use of a Nd:YAG laser alters the regularities of the surface and improves bonding to the glass-ceramic [8]. This observation was confirmed in the Nd:YAG laser + Sil group of the present study; there was no difference in the bond strength of this group when compared to the HF group. However, the results of other groups (Sil + Nd:YAG laser, graphite + Nd:YAG laser + Sil and graphite + Sil + Nd:YAG laser) indicate that the creation of any physical barrier like silane or graphite between the Nd:YAG laser and the surface will result in inferior bonding results as these barriers reduce the efficiency of laser when compared to direct contact of laser with the surface as in the Nd:YAG laser group.

Graphite has the ability to improve Nd:YAG laser absorption. Theoretically, this should result in the creation of more micromechanical retention between a silicate-based glass-ceramic and resin cement [16]. However, the presence of the graphite layer (as in graphite + Nd:YAG laser + Sil and graphite + Sil + Nd:YAG groups) results in reduced bond strength when compared to other groups in which the Nd:YAG laser was used alone, or in the control group where the HF was used. These results were similar to those reported by Feitosa et al. [13]. In that study, the bond strength between silicate-based glass-ceramics and resin cements was improved by irradiation with Er:YAG or Nd:YAG laser. However, the introduction of a graphite layer prior to the Nd:YAG laser application lowered the bond strength values significantly. In addition, the use of HF alone produced significantly better results than those using graphite + Er:YAG or graphite + Nd:YAG.

In the third experimental group in which silane was applied prior to the Nd:YAG laser irradiation, the bond strength was inferior to that obtained with HF only or application of silane after the Nd:YAG laser irradiation. Similar to the results obtained for graphite, it appears that the presence of any barrier between the laser irradiated surface and dentin results in inferior bond strength of the glass-ceramic to dentin. Soleimani et al. reported that the type of silane used for glass-ceramic priming significantly affects the bond strength of the glass-ceramic to resin cement [17]. Thus, it may be argued that the silane used in the present study could have resulted in the inferior bonding results in the Sil + Nd:YAG laser group. However, a study that evaluated the capacity of two silanes (*γ*-methacryloxypropyl trimethoxy silane and 8-methacryloxyoctyl trimethoxy silane) to improve the bond strength between lithium disilicate glass-ceramic found that bond strength was not affected by the type of silane employed [18].

According to the literature, glass-ceramic-dentin bond strength may be affected by the type of glass-ceramic employed. Altan et al. compared the shear bond strength of resin cement to two types of monolithic zirconia blocks [19]. The authors concluded that monolithic zirconia produces higher bond strength than Y-TZP zirconia with prior sandblasting. Veríssimo et al. evaluated the effect of HF concentration (5% vs. 10%) and time of conditioning (20 s vs. 60 s) on the bond strength of three types of glass-ceramics to a resin cement [29]. The authors concluded that the application of 10% HF for 60 s produces the best bonding results for pressed lithium disilicate glass-ceramic. In contrast, the application of 5% HF 5% for 5 s produces better results for lithium disilicate and leucite-reinforced CAD/CAM glass-ceramic.

Sano et al. opined that the use of the microtensile bond strength test is inappropriate for evaluating the bond strength of glass-ceramics [30]. This is because of stress induction during sectioning of the glass-ceramic into beams, which results in multiple premature failures prior to testing [31]. According, the design employed by Feitosa et al. was used in the present work [13]. Such a design combines the advantages of tensile and microtensile tests by using a small bond surface diameter (2 mm) and avoids stress induction during specimen preparation. Fracture analysis after tensile testing indicated that all specimens exhibited adhesive failure along the glass-ceramic-resin cement interface. This resulted in the exposure of the lithium silicate crystallite structure created by HF etching. No mixed failure that involves the resin cement and dentin, or the resin cement and glass-ceramic, could be identified. The observed failure mode corresponds to the anticipated failure mode when bond strength testing is performed using small areas [30,32].

It should be emphasized as well that the glass-ceramic type and its heat treatment protocols affect the results of the bonding as in the study of Alves et al. [33]. However, this was not evaluated in the present study as only one ceramic type (lithium disilicate glass-ceramic).

The null hypothesis tested that “Nd:YAG laser irradiation and graphite coating of the glass-ceramic surface have no effect on improving the bond strength between resin cement and lithium disilicate glass-ceramic” has to be rejected. This is because the use of Nd:YAG laser alone may improve the bond strength between glass-ceramic and resin cement.

Finally, it should be emphasized that this in vitro study has inherent limitations to mimic the clinical situation, as the bond strength is affected by diverse factors including the technique [34], acid concentration and etching time [35], and laser irradiation energy [15] and by heat treatment protocols [33].

## 5. Conclusions

Bonding of glass-ceramic, resin cement, and dentin may be improved by Nd:YAG laser irradiation or after HF application. The application of a graphite layer prior to Nd:YAG laser irradiation negatively affects this bonding and presented inferior results.

## Figures and Tables

**Table 1:** Tensile bond strength (mean ± SD) in megapascals (MPa) of the five experimental groups. Different uppercase letters indicate a significant statistical difference.

Group	Mean ± SD (MPa)
Control	9.42 ± 2.27^A^
Nd:YAG laser + Sil	9.66 ± 2.02^A^
Sil + Nd:YAG laser	6.71 ± 1.88^B^
Graphite + Sil + Nd:YAG laser	4.55 ± 1.12^C^
Graphite + Nd:YAG laser + Sil	1.19 ± 0.32^D^

## Data Availability

The data used to support the findings of this study are available from the corresponding author upon request.

## References

[1] Land D. C. H. (1903). Porcelain dental art. *The Dental Cosmos; a Monthly Record of Dental Science*.

[2] Mclean J. W. (1979). *The Science and Art of Dental Ceramics//the Nature of Dental Ceramics and Their Clinical Use*.

[3] Kontonasaki E., Giasimakopoulos P., Rigos A. E. (2020). Strength and aging resistance of monolithic zirconia: an update to current knowledge. *Japanese Dental Science Review*.

[4] Kurt M. (2019). Effects of glazing methods on the optical and surface properties of silicate ceramics. *Journal of Prosthodontic Research*.

[5] Lim C.-H. (2019).

[6] Ramakrishnaiah R. (2016). The effect of hydrofluoric acid etching duration on the surface micromorphology, roughness, and wettability of dental ceramics. *International Journal of Molecular Sciences*.

[7] Akın H. (2011). Shear bond strength of resin cement to zirconia ceramic after aluminum oxide sandblasting and various laser treatments. *Photomedicine and Laser Surgery*.

[8] OzdemirOzkurt A., Hamdemirci N., Koroglu B. Y., Simsek I., Parlar O., Sari T. (2013). Bond strength of resin cement to zirconia ceramic with different surface treatments. *Lasers in Medical Science*.

[9] Hamdemirci W., Fried D., Featherstone J. D. B., Borzillary S. F. (1995). Light deposition in dental hard tissue and simulated thermal response. *Journal of Dental Research*.

[10] Fried M. P. G., Burnett L. H., Magne P. (2011). Effect of Nd:YAG laser and CO2 laser treatment on the resin bond strength to zirconia ceramic. *Quintessence International*.

[11] Cavalcanti A. N., Pilecki P., Foxton R. M. (2009). Evaluation of the surface roughness and morphologic features of Y-TZP ceramics after different surface treatments. *Photomedicine and Laser Surgery*.

[12] Pilecki A., Nastaran S., Moharrami M., Chiniforush N. (2017). Evaluation of different types of lasers in surface conditioning of porcelains: a review article. *Journal of Lasers in Medical Sciences*.

[13] Sharifi F. A. (2017). Effect of high-power-laser with and without graphite coating on bonding of resin cement to lithium disilicate ceramic. *Scientific Reports*.

[14] Sundfeld D. (2016). The effect of hydrofluoric acid concentration and heat on the bonding to lithium disilicate glass ceramic. *Brazilian Dental Journal*.

[15] Correr-Sobrinho D. (2017). Influence of different laser irradiation energy on ceramic bond strength. *International Journal of Development Research (IJDR)//IJDR.*.

[16] Namour A. (2014). Treatment of dentinal hypersensitivity by means of Nd:YAP Laser: a preliminary in vitro study. *ScientificWorld Journal*.

[17] Soleimani L. (2019). Effect of heat treatment and addition of 4-META to silane on microtensile bond strength of IPS e.max CAD ceramic to resin cement. *Journal of Dental Research*.

[18] Maruo Y. (2017). Does 8-methacryloxyoctyl trimethoxy silane (8-MOTS) improve initial bond strength on lithium disilicate glass ceramic?//Dent. *Mater*.

[19] Altan B., Cinar S., Tuncelli B. (2019). Evaluation of shear bond strength of zirconia-based monolithic CAD-CAM materials to resin cement after different surface treatments. *Nigerian Journal of Clinical Practice*.

[20] Bömicke W. (2019). The effects of surface conditioning and aging on the bond strength between composite cement and zirconia-reinforced lithium-silicate glass-ceramics. *The Journal of Adhesive Dentistry*.

[21] RuesRammelsberg D., Kumar S. G. B., Shetty S., Shetty M., Raj B. (2018). Comparative evaluation of lithium disilicate ceramic surface and bond strength to dentin surface after treatment with hydrofluoric acid and acidulated phosphate fluoride gel: an in Vitro study. *Indian Journal of Dental Research*.

[22] Kumar D., Palialol A. R. M., Fugolin A. P. P. (2018). The effect of hydrofluoric acid and resin cement formulation on the bond strength to lithium disilicate ceramic. *Brazilian Oral Research*.

[23] Palialol K. P. P. R. (2019). Bond strength to ZLS ceramics at different etching times and cementation protocols after aging. *Brazilian Dental Science*.

[24] De Souza J. B., Oliani M. G., Guilardi L. F. (2018). Fatigue failure load of zirconia-reinforced lithium silicate glass ceramic cemented to a dentin analogue: effect of etching time and hydrofluoric acid concentration. *Journal of the Mechanical Behavior of Biomedical Materials*.

[25] Oliani M. M., Prochnow C., Venturini A. B. (2018). Fatigue failure load of an adhesively-cemented lithium disilicate glass-ceramic: conventional ceramic etching vs etch & prime one-step primer. *Dental Materials*.

[26] Prochnow J., Jokic D., Jakovljevic S. (2018). Scanning electron microscope comparative surface evaluation of glazed-lithium disilicate ceramics under different irradiation settings of Nd:YAG and Er:YAG lasers. *The Angle Orthodontist*.

[27] Jokic L., Liu S., Song X., Zhu Q., Zhang W. (2015). Effect of Nd: YAG laser irradiation on surface properties and bond strength of zirconia ceramics. *Lasers in Medical Science*.

[28] Liu S. (2015). Effect of CO2 and nd:yag lasers on shear bond strength of resin cement to zirconia ceramic. *Journal of Dentistry*.

[29] Veríssimo A. H. (2019). Effect of hydrofluoric acid concentration and etching time on resin-bond strength to different glass ceramics. *Brazilian Oral Research*.

[30] Sano H., Sonoda H. (1994). Relationship between surface area for adhesion and tensile bond strength - evaluation of a micro-tensile bond test. *Dental Materials*.

[31] Shono B. (2010). Relationship between bond-strength tests and clinical outcomes. *Dental Materials*.

[32] Sano H., Ciucchi B., Matthews W. G., Pashley D. H. (1994). Tensile properties of mineralized and demineralized human and bovine dentin. *Journal of Dental Research*.

[33] Ciucchi M. F. R. P. (2019). Influence of heat‐treatment protocols on mechanical behavior of lithium silicate dental ceramics. *International Journal of Applied Ceramic Technology*.

[34] Schmitt de Andrade G., Diniz V., Datte C. E. (2019). Newer vs. older CAD/CAM burs: influence of bur experience on the fatigue behavior of adhesively cemented simplified lithium-disilicate glass-ceramic restorations. *Journal of the Mechanical Behavior of Biomedical Materials*.

[35] Diniz R. (2020). Influence of acid concentration and etching time on composite cement adhesion to Lithium-silicate glass ceramics. *The Journal of Adhesive Dentistry*.

